# Proteomic investigations of adult polyglucosan body disease: insights into the pathobiology of a neurodegenerative disorder

**DOI:** 10.3389/fneur.2023.1261125

**Published:** 2023-11-14

**Authors:** Joseph R. Abraham, Frederick M. Allen, John Barnard, Daniela Schlatzer, Marvin R. Natowicz

**Affiliations:** ^1^Cleveland Clinic Lerner College of Medicine of Case Western Reserve University, Cleveland, OH, United States; ^2^Department of Quantitative Health Sciences, Lerner Research Institute, Cleveland Clinic, Cleveland, OH, United States; ^3^Center for Proteomics, Case Western Reserve University School of Medicine, Cleveland, OH, United States; ^4^Pathology and Laboratory Medicine, Genomic Medicine, Neurological and Pediatrics Institutes, Cleveland Clinic, Cleveland, OH, United States

**Keywords:** adult polyglucosan body disease, *GBE1*, glycogen branching enzyme, glycogen storage disease, neurodegeneration, pathogenesis, proteomic, lymphoblast

## Abstract

Inadequate glycogen branching enzyme 1 (GBE1) activity results in different forms of glycogen storage disease type IV, including adult polyglucosan body disorder (APBD). APBD is clinically characterized by adult-onset development of progressive spasticity, neuropathy, and neurogenic bladder and is histologically characterized by the accumulation of structurally abnormal glycogen (polyglucosan bodies) in multiple cell types. How insufficient GBE1 activity causes the disease phenotype of APBD is poorly understood. We hypothesized that proteomic analysis of tissue from GBE1-deficient individuals would provide insights into GBE1-mediated pathobiology. In this discovery study, we utilized label-free LC–MS/MS to quantify the proteomes of lymphoblasts from 3 persons with APBD and 15 age- and gender-matched controls, with validation of the findings by targeted MS. There were 531 differentially expressed proteins out of 3,427 detected between APBD subjects vs. controls, including pronounced deficiency of GBE1. Bioinformatic analyses indicated multiple canonical pathways and protein–protein interaction networks to be statistically markedly enriched in APBD subjects, including: RNA processing/transport/translation, cell cycle control/replication, mTOR signaling, protein ubiquitination, unfolded protein and endoplasmic reticulum stress responses, glycolysis and cell death/apoptosis. Dysregulation of these processes, therefore, are primary or secondary factors in APBD pathobiology in this model system. Our findings further suggest that proteomic analysis of *GBE1* mutant lymphoblasts can be leveraged as part of the screening for pharmaceutical agents for the treatment of APBD.

## 1 Introduction

The glycogen biosynthetic enzyme gene *GBE1* encodes the glycogen branching enzyme (EC 2.4.1.18), an enzyme that catalyzes the transfer of alpha-1,4 linked glucosyl units from the outer end of a glycogen chain to an alpha-1,6 position on the same or nearby oligosaccharide chain. This enzyme activity results in the branching of high molecular weight glycogen molecules enabling the packing of a large number of glycosyl units into a relatively soluble spherical molecule (1). The absence or defective function of glycogen branching enzyme results in one of several clinical forms of autosomal recessive glycogen storage disease type IV (GSD type IV; OMIM #232500) in humans and in mice and other animal models (1). Despite marked heterogeneity in age of onset and natural history, all clinical forms of GSD type IV are associated with diminished or absent glycogen branching enzyme activity and an accumulation of structurally abnormal glycogen in tissues.

Adult polyglucosan body disease (APBD) is an allelic form of GSD type IV that is characterized by the progressive development of spastic paraparesis, neurogenic bladder and peripheral neuropathy, usually beginning in the 5^th^ or 6^th^ decade of life (2, 3). APBD is associated with the deposition of a poorly soluble form of glycogen, polyglucosan bodies, in multiple tissues; its accumulation in the central and peripheral nervous system correlates with the major clinical findings (2, 3). Most persons that have been diagnosed with APBD are of Ashkenazi Jewish ethnicity and are homozygous for the *GBE1* c.986A > C, p.Y329S mutation or have compound heterozygosity for that allele and a *GBE1* c.2053-3358_2053-3350delinsTGTTTTTTACATGACAGGT intronic mutation (4).

The underlying pathogenetic basis of APBD is only partially understood. There are abundant data regarding the cytological abnormalities in diverse cells and tissues in ABPD and other forms of GSD-IV based on light and electron microscopic analyses of autopsy- and biopsy-derived tissues from affected individuals that provide some insights into disease pathobiology (1, 5). The development and characterization of a murine model of APBD that recapitulates this form of GSD-IV has allowed detailed histological characterization of multiple tissues across the lifespan and enabled important investigations (6). However, much remains to be learned regarding how the downstream molecular consequences of GBE1 enzyme deficiency in APBD cause cell type-specific processes and the associated tissue and organ dysfunction.

Lymphocytes show pathologic polyglucosan body accumulation in APBD, making study of these cells a potentially useful experimental system to investigate the biology of this condition (7). To gain insight into the pathobiology of APBD, we utilized a proteomic approach to identify differentially expressed proteins and differentially expressed pathways and protein–protein interaction networks between lymphoblasts from persons having APBD and matched controls. Proteomic analyses of tissues and of single cells have been a powerful tool to discover and elucidate biochemical pathways, networks and processes in normal and pathologic states of many organisms (8, 9). In this report, we identify numerous differentially expressed proteins, cellular pathways and protein–protein networks of pathologic significance in APBD. In so doing, we provide support for several pathogenetic mechanisms that have recently been proposed and provide evidence suggesting still other pathogenetic processes. The data further suggest the potential application of this system in screening pharmaceutical agents for the treatment of APBD.

## 2 Materials and methods

### 2.1 Specimens

Lymphoblastoid cell lines were developed from peripheral blood samples from three male subjects, ages 63, 64, and 70 years, and 15 healthy age- and gender-matched controls. The three subjects were of Ashkenazi Jewish ethnicity and had clinical and neuroimaging findings characteristic of APBD for persons of their ages. The subjects were compound heterozygotes for the *GBE1* DNA sequence variants c.986A > C, p.Y329S and *GBE1* c.2053-3358_2053-3350delinsTGTTTTTTACATGACAGGT (4). The c.986A > C variant is classified as pathogenic using the 2015 ACMG/AMP variant classification criteria [(10); criteria PS3, PM3, PP1, PP3]. The *GBE1* c.2053-3358_2053-3350delinsTGTTTTTTACATGACAGGT variant is also classified as pathogenic when the 2015 ACMG/AMP variant classification criteria are applied (criteria PS3, PM3, PP3). The protocol for this work was approved by the Cleveland Clinic Institutional Review Board and informed consent was provided by each participant.

### 2.2 Lymphoblast cell culture conditions

Human peripheral blood mononuclear cells were harvested and transformed with the Epstein–Barr virus as described (11). The immortalized lymphoblasts were cultured and passaged in RPMI-1640 (Life Technologies) supplemented with heat-inactivated fetal bovine serum (Gemini Bio Products, 20% final volume), antibiotic-antimycotic solution (Life Technologies, 1X final volume) and Tylosin solution (Sigma-Aldrich, 0.013 mg/mL final volume) into three T75 culture flasks. After becoming confluent the cells from the individual flasks were harvested via centrifugation at 600 × *g* for 10 min at room temperature. They were then washed in 30 mL of 1X PBS and an aliquot of each was set aside for counting via dye exclusion. The cells were centrifuged again at 600 × *g* for 10 min at room temperature and the supernatant decanted. The cell pellets were transferred to a 1.8 mL microfuge tube, quickly spun and any residual PBS removed. The resultant cell pellets were then stored at −80°C until further use.

### 2.3 Unbiased label-free LC–MS/MS proteomic analysis

Lymphoblast samples were lysed with 2% SDS/0.1% protease inhibitor cocktail (Sigma-Aldrich, St. Louis, MO), then sonicated, followed by filter-aided sample preparation detergent clean-up. 15 mcg protein of each sample were digested with LysC for 1 h and trypsin overnight at 37°C. Reverse phase LC–MS/MS was performed as described (12). All samples were processed identically and at the same time and the order of their chromatographic positions randomized.

LC–MS/MS data were processed using Rosetta Elucidator (Rosetta Biosoftware, Seattle, WA) (Version 3.3.01 SP4 25), Mascot (version 2.4.1) (Matrix Science, London, UK) and the human Uniprot database. Automated differential quantification and identification of peptides was performed as previously described (13, 14). The mass spectrometry proteomics data have been deposited to the ProteomeXchange Consortium via the PRIDE (15) partner repository with the dataset identifiers PXD012558 and 10.6019/PXD012558.

### 2.4 Statistical methods

Raw MS data were obtained and missing values were then imputed using a weighted k-nearest neighbors method (16). Data were then log_2_-transformed and preprocessing steps performed using InfernoRDN (17).

To increase statistical power, we treated individual peptides as observations of a given protein. Data were imported into the R statistical programming environment for subsequent analyses. Using the lme4 package (18), a linear mixed effects model was applied to each protein with the following form:


$$\mathrm{intensity}=\beta_{1}.\mathrm{CONDITION}+\beta_{2}.\mathrm{AGE}+\beta_{3}.\mathrm{PEPTIDE}+\epsilon$$

where intensity refers to the log_2_-transformed intensity values for each peptide observed for that protein, condition a binomial categorical variable (APBD-subject or Control), age a continuous variable, peptide a multi-level random effects categorical variable with the number of levels dependent on the protein, and ϵ is independent normally-distributed residual error with mean 0 and standard deviation σ. A likelihood ratio was constructed for each coefficient in the model to assess its contribution to the variance in the data, and *p*-values were obtained. *P*-values were adjusted using the false discovery rate method described by Benjamini and Hochberg (19).

### 2.5 Bioinformatic analyses

Proteins identified by only one unique peptide were excluded from differential expression analysis. Network and pathway analyses were performed with the Ingenuity Pathway Analysis^®^ software (IPA)^1^; proteins with Benjamini-Hochberg adjusted *p*-value < 0.05 were used for this purpose. Enrichment scores and *p*-values for canonical pathway were determined by a one-tailed Fisher’s exact test using the complete Ingenuity Knowledge Base as a reference. Canonical pathway *p*-values were further adjusted for multiple comparisons using the Benjamini-Hochberg procedure.

### 2.6 Targeted MS analysis with selected reaction monitoring

Validation of the label-free global proteomic method was done through selected reaction monitoring (SRM) MS to quantify the levels of several differentially expressed proteins noted in the label-free (unbiased or ‘shotgun’) proteomic analysis. The criteria for peptide selection for SRM MS were differential expression in the label-free LC–MS/MS analysis and biological interest of the proteins to which those peptides belong. Targeted MS analyses were performed as described previously using synthesized deuterium-labeled peptides as internal standards for quantification (Sigma-Aldrich, St. Louis, MO) (20). Coefficients of variation (CVs) for the 7 peptides used in the SRM analyses, as well as 6 additional deuterium-labeled peptides, were calculated after 34 LC–MS/MS analyses; the CVs ranged from 8.57–14.08%, with a mean of 11.30%. Analyses of the peptides between the untargeted and targeted analyses were done to determine whether the differential expression seen in the untargeted remained statistically significant using one-tailed Wilcoxon signed-rank tests. In addition, we determined the concordance of the directionality and magnitude of expression between the targeted and untargeted data.

## 3 Results

### 3.1 Protein identification and differential protein expression in lymphoblasts

The LC–MS/MS analysis of APBD and control samples detected 15,792 peptides belonging to 3,426 proteins. Of the proteins detected in APDB subjects and controls, 68.6% of proteins (2351) were identified by at least two uniquely identifying peptides; the latter comprised the set of proteins for further study because of the increased accuracy of the quantification when at least 2 peptides are used to quantify proteins by the LC–MS/MS method. An analysis of proteins identified by ≥2 unique peptides revealed 531 proteins having significant differential expression between APBD and controls (adjusted *p*-value < 0.05). Table 1 notes the top 30 differentially expressed proteins identified by ≥2 peptides with adjusted *p*-values and percent expression changes. The most statistically significant differentially expressed protein was GBE1 (9.3% in APBD subjects vs. controls; adjusted *p*-value 5.87E-40), an important ‘positive control.’ The top 30 and the entire set of 531 differentially expressed proteins are detailed in Table 1 and Supplementary Table 1, respectively.

**Table 1:** Top differentially expressed proteins between APBD subjects vs. controls.

Protein	Expression, % (APBD vs. Controls)	*p*-value	Adjusted *p*-value
1,4-alpha-glucan-branching enzyme	9.3	2.50E-43	5.87E-40
Ribosome-binding protein 1	187.0	3.63E-30	4.27E-27
Beta-II spectrin	79.1	6.23E-23	4.88E-20
Brain-type aldolase	62.1	5.67E-20	3.33E-17
Alpha-II spectrin	84.8	1.49E-19	7.00E-17
Ran GTPase-activating protein 1	141.9	5.67E-18	2.22E-15
UDP-Glc dehydrogenase	173.9	1.31E-16	4.42E-14
Ena/VASP-like protein	64.3	1.56E-16	4.57E-14
Talin-1	110.5	2.91E-16	7.60E-14
Importin-5	118.1	1.34E-15	3.15E-13
Adenosine deaminase	65.0	2.57E-15	5.48E-13
p100 co-activator	117.3	2.92E-13	5.73E-11
Vimentin	193.6	1.01E-12	1.83E-10
MARCKS-like protein 1	43.7	1.29E-12	2.17E-10
FPP synthase	71.3	1.99E-12	3.12E-10
Nampt	75.0	2.48E-12	3.64E-10
Tripeptidyl-peptidase 2	120.5	6.31E-12	8.72E-10
Nuclear factor NF-kappa-B p100 subunit	65.9	1.10E-11	1.44E-09
Astrocyte elevated gene-1 protein	124.3	1.18E-11	1.46E-09
Immunoglobulin heavy constant mu	232.3	2.63E-11	3.09E-09
eIF-2-alpha kinase activator GCN1	112.5	7.86E-11	8.80E-09
Raftlin	74.1	1.21E-10	1.23E-08
Hypoxia up-regulated protein 1	133.1	1.17E-10	1.23E-08
Endoplasmin	125.6	1.38E-10	1.35E-08
Ribonucleoside-diphosphate reductase large subunit	125.8	1.67E-10	1.57E-08
Proliferating cell nuclear antigen	139.9	2.86E-10	2.58E-08
DNA replication licensing factor MCM2	117.3	4.52E-10	3.94E-08
Fascin	71.4	4.80E-10	4.03E-08
Vasodilator-stimulated phosphoprotein	78.8	2.11E-09	1.71E-07
DNA replication licensing factor MCM5	121.2	2.19E-09	1.71E-07

*P*-values were adjusted for multiple comparisons using the Benjamini-Hochberg procedure.

The average and median coefficients of variation (CV) for all of the peptides used for quantification of proteins were determined and the mean CVs for subjects and controls were identical, as were the median CVs for subjects and controls (Supplementary Table 2). The mean and median CV data, revealing an average CV below 20%, indicate a high degree of consistency in protein expression among subjects and among controls. In addition, analysis of the levels of the differentially expressed proteins indicates that only a small subset of the differentially expressed proteins – exemplified by GBE1 – have markedly different levels between the subjects and controls. Specifically, just 23 of the 531 (4.3%) differentially expressed proteins of the subjects showed levels either 50% less or 50% greater than the levels of those proteins in controls.

### 3.2 Canonical pathways and network analyses

To more fully understand the meanings of the differentially expressed proteins, we used the Ingenuity Pathway Analysis ^®^ (IPA) bioinformatics tool to determine whether specific pathways are enriched in APBD subjects vs. controls. We found enrichment in APBD subjects for 33 pathways at adjusted *p*-value < 0.05 after adjusting for multiple comparisons using the Benjamini-Hochberg (BH) procedure. Amongst the findings of greatest significance were pathways relating to translation, mTOR signaling, protein ubiquitination, unfolded protein response, glycolysis, endocytosis and cell death (Table 2).

**Table 2:** Top canonical pathways from differentially expressed proteins (*p* < 0.05), adjusted for multiple comparisons.

Canonical pathways	Enrichment	Canonical pathways	Enrichment
EIF2 signaling	18.3	Tumoricidal function of hepatic natural killer cells	2.26
Regulation of eIF4 and p70S6K signaling	17.7	Granzyme B signaling	1.98
mTOR signaling	13.6	Sumoylation pathway	1.98
Protein ubiquitination pathway	10.4	Clathrin-mediated endocytosis signaling	1.98
Unfolded protein response	5.26	Huntington’s disease signaling	1.66
tRNA charging	4.7	Virus entry via endocytic pathways	1.6
Cell cycle control of chromosomal replication	4.32	Endoplasmic reticulum stress pathway	1.6
RAN signaling	4.02	Apoptosis signaling	1.6
Glycolysis I	4.02	Actin nucleation by ARP-WASP complex	1.44
Aldosterone signaling in epithelial cells	3.87	Role of CHK proteins in cell cycle checkpoint control	1.41
Caveolar-mediated endocytosis signaling	3.54	ATM signaling	1.41
Pyrimidine deoxyribonucleotides *de novo* biosynthesis I	3.32	Mechanisms of viral exit from host cells	1.38
Death receptor signaling	2.58	Mitochondrial dysfunction	1.38
Purine nucleotides *de novo* biosynthesis II	2.53	ILK signaling	1.38
BER pathway	2.39	Lipid antigen presentation by CD1	1.36
Mitotic roles of polo-like kinase	2.32	Glutathione redox reactions II	1.36
PI3K/AKT signaling	2.26		

An enrichment score of 1.3 equals an adjusted *p*-value of 0.05.

**Figure 1:**
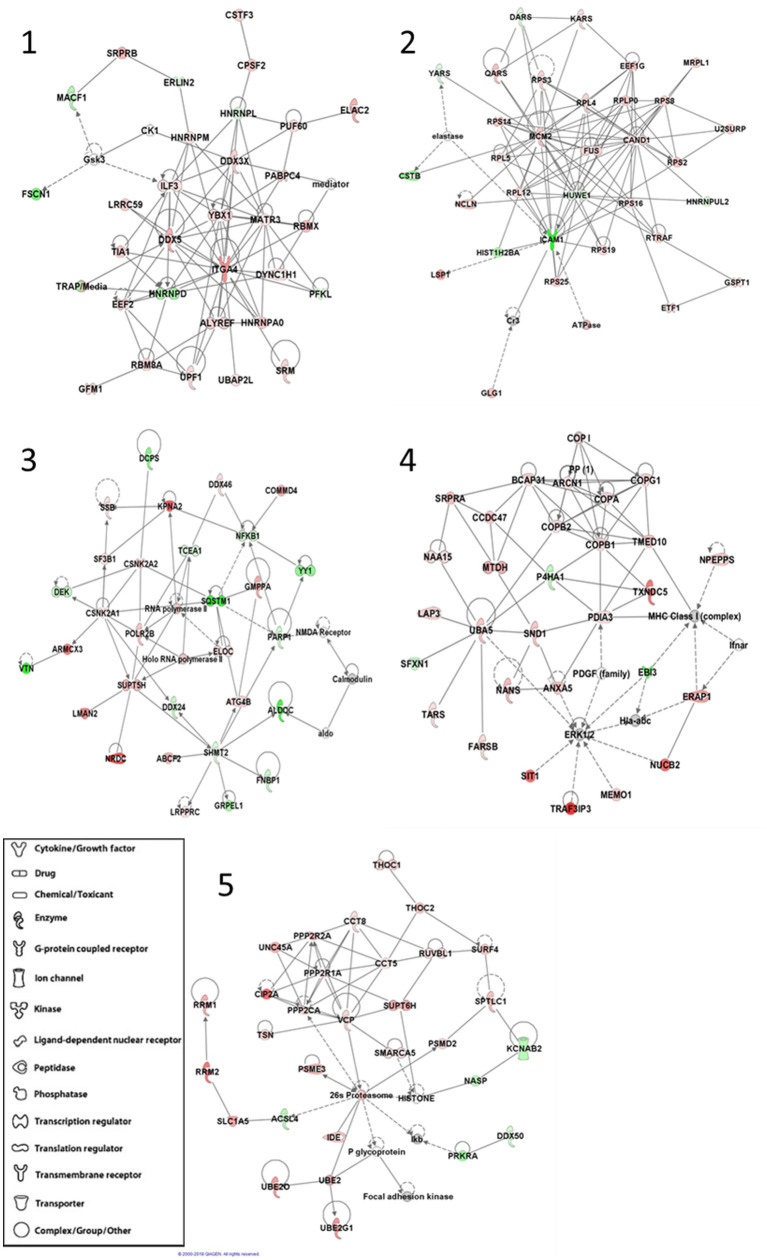
IPA^®^-generated statistically significant protein–protein interaction networks. Each protein in the interaction networks is depicted by a shape that corresponds to its function or identity and overlayed by the protein name or abbreviation. Proteins are connected within the networks by lines that indicate either a direct (solid) or indirect (dotted) relationship. Green proteins are down-regulated in APBD-subjects relative to controls, while red proteins are up-regulated.

We also sought to determine any protein–protein interaction networks that might also be differentially expressed between APBD subjects and controls. After inputting the differentially expressed proteins, IPA software determined five highly significant interaction networks (Figure 1). Four of the five networks highlighted biological themes relating to multiple aspects of transcription and translation (networks 1, 2, 3, 5), including transcriptional regulation, splicing and transport of mRNAs, and ribosomal and tRNA charging functions. Biological themes of the chaperoning of proteins, protein transport, protein ubiquitination, or proteasomal degradation were enriched in four networks (networks 2, 3, 4, 5); major hub molecules involved in these functions include HUWE1, CAND1, SQSTM1, UBA5, VCP, and the 26 s proteasome. Diverse intracellular organelle dynamics including autophagy and ER and Golgi system structure/function are highlighted in network 3 (hub proteins SQSTM1, ATG4B) and network 4 (hub proteins ARCN1, PDIA3, BCAP31 and multiple coatomer proteins), respectively. Other highlighted cellular processes include cell cycle regulation/apoptosis (networks 2, 3) and some metabolic functions such as serine/glycine metabolism and glycolysis (network 3). Predicted upstream regulators that drive the differential expression between APBD subjects and controls, based on IPA analysis, are especially enriched in transcription regulators (e.g., MYC, TP53, XBP1) and several cytokines (Supplementary Table 3).

### 3.3 Validation analysis with selective reaction monitoring mass spectrometry

To independently validate and confirm the unbiased label-free proteomic findings, we performed a targeted quantitative MS analysis, selected reaction monitoring (SRM), of uniquely identifying peptides for selected proteins of interest, comparing both the directionality of expression and the levels of the peptides noted in the unbiased vs. targeted analysis. All 7 peptides selected for SRM validation analysis, from 4 proteins of interest, showed concordance in the direction of expression (i.e., increased or decreased expression of APBD subjects relative to controls), with similar percent expression between the unbiased, label-free and targeted proteomic analyses (Table 3). Moreover, each protein that was represented by 2 peptides in the SRM analysis and for which a “protein level” comparison therefore could be made was differentially expressed at the protein level statistically (GBE1, RRBP1, ALDOC), further corroborating the label-free analysis.

**Table 3:** Summary of targeted MS validation analysis using one-sided Wilcoxon signed rank tests.

APBP						
Protein	Peptide	Directionality concordance	% Expression A vs. C (global)	% Expression A vs. C (SRM)	Global *q*-value	SRM *p*-value
GBE1	–	✓	10	19	5.87E-40	0.0156
	YGWLAAPQAYVSEK	✓	–	17	–	0.125
	IVLDSDAAEYGGHQR	✓	–	21	–	0.125
RRBP1	–	✓	189	141.5	4.27E-27	0.0156
	LLATEQEDAAVAK	✓	–	147	–	0.125
	TLQEQLENGPNTQLAR	✓	–	136	–	0.125
ALDOC	–	✓	65	76.5	3.33E-17	0.0156
	DDNGVPFVR	✓	–	82	–	0.125
	DNAGAATEEFIK	✓	–	71	–	0.125
EVL	QVQNGPSPDEMDIQR	✓	65	46	4.57E-14	0.125

Directionality concordance assessed whether differential expression aligned with global proteomic findings at both the peptide and protein levels. *P*-values evaluating protein concentrations between cases and controls cannot be meaningfully determined for the samples that had one peptide for paired analysis.

## 4 Discussion

GBE1 is a glycogen branching enzyme that catalyzes the transfer of alpha-1,4-linked glucosyl units to an alpha-1,6 position on the same or adjacent glycogen chain. Branching of glycogen chains is important for the synthesis of structurally normal glycogen. The absence or a critical insufficiency of GBE1 activity results in the accumulation of structurally abnormal, poorly soluble glycogen and one of the clinical forms of autosomal recessive glycogen storage disease type IV (1).

Adult polyglucosan body disease (APBD) represents the “mildest” known clinical form of GSD IV although it is, nevertheless, a neurodegenerative condition associated with significant and progressive central and peripheral nervous system sequelae (2, 3). The molecular basis of the underlying disease process in APBD is inadequately understood. Here, we sought to leverage proteomic methodology to obtain additional insights regarding the molecular basis of APBD pathogenesis. Using an unbiased label-free LC–MS/MS approach we identified 531 lymphoblast proteins that were significantly differentially expressed between APBD subjects and controls and multiple metabolic pathways and protein–protein interaction networks that were markedly differentially expressed between APBD and controls.

Determination of the primary pathogenetic mechanism(s) in APBP presents significant challenges. Elucidation of the pathophysiology is complex for several reasons. First, there are varying glycogen biosynthetic and degradative capacities in different cell types, including within the central nervous system (CNS). Related to this and illustrating the complexity, recent studies reveal molecular heterogeneity of soluble and insoluble glycogen in GBE1-deficient cells and demonstrate that different cell types can produce distinct types of polyglucosan bodies and that there can be variation of the storage product even within a specific cell type (21–23). Second, there is evidence of varied cytological sensitivity to the accumulation of polyglucosan bodies across different cell types and tissues. Third, despite extensive study, there is an incomplete understanding of the functions of glycogen in different cell types of the CNS (24–26).

Hypotheses of ABPD disease pathogenesis include the non-mutually exclusive concepts of bioenergetic dysfunction resulting from an insufficiency of structurally normal glycogen and cellular toxicities secondary to the accumulation of the structurally abnormal polyglucosans. The determination of the basis of ABPD pathogenesis is important for the development of effective therapeutic approaches (27–33).

Histological and electron microscopic analyses of tissues from GBE1-deficient individuals and animal models of APBD have provided some basic, important information about the pathogenesis of APBD. Investigations of tissues from persons with APBD have shown varied polyglucosan accumulation in multiple tissue and cell types, including throughout much of the CNS, peripheral nerves, skeletal muscle, diaphragm, heart, liver, lungs, kidneys, sweat glands and some immune cells (7, 33–37), with well-documented variability of involvement of the same tissues even in persons having identical *GBE1* mutant genotypes (23). A widely used mouse model of APBD created by homozygous knock-in of the most common APBD mutation in humans showed a progressive neuromuscular decline phenotype with polyglucosan body accumulation noted in multiple tissues, including non-neuronal tissues (6). The data from these human and animal model studies clearly indicate that polyglucosan body accumulation does not cause clinically apparent involvement of all tissues having this histologic finding and that the underlying pathophysiology related to polyglucosan body accumulation is tissue and cell type specific, with each tissue “telling its own clinical story.”

One important study emphasized the central significance of polyglucosan body accumulation in human astrocytes in the pathophysiology of APBD. The authors proposed, based on current understanding of glycogen metabolism in astrocytes and neurons and their metabolic intercellular coupling, that the accumulation of glycogen in astrocytes due to GBE1 deficiency likely results in a lack of energy substrates, first for astrocytes and then for neurons, and is consistent with a bioenergetic deficiency caused by GBE1 deficiency (37). Structurally abnormal muscle mitochondria have also been noted in individuals with APBD (35). In other work, human fibroblasts from APBD subjects showed reduced mitochondrial biomass, mitochondrial membrane depolarization and reduced mitochondrial oxidative phosphorylation and suggested variably increased glycolytic ATP production when grown in conditions maximizing glycogen burden (33).

Analyses of the above mouse model of APBD add to this theory of APBD pathogenesis. The adult mutant mice show mildly low serum glucose levels (6, 33). In addition, the mutant adult mice have a lower respiratory quotient, total energy expenditure and fat oxidation compared to wild type controls (33). While frank hypoglycemia is not a recognized clinical concern for humans with ABPD, biochemical abnormalities consistent with a secondary energy deficit have been noted (27). Our proteomic analysis showed a significant enrichment for mitochondrial dysfunction in APBD lymphoblasts, amongst several prominent cellular themes (Table 2). Overall, then, there is now compelling evidence from both human studies and mouse models of APBD in support of a secondarily-induced disturbance of bioenergetics in different cell types in APBD.

Some light and electron microscopic evaluations of tissues from individuals with APBD have shown polyglucosan body accumulation within membranous structures, identified as autophagosomes (7), although most polyglucosan bodies are not membrane delimited. In another study, alleviation of polyglucosan storage in human APBD fibroblasts by a specific drug, compound 144DG11, was associated with enhancement of autophagocytic glycogen catabolism (33). Other work demonstrated ubiquitination of human APBD muscle polyglucosan bodies and of APBD mouse liver, heart and skeletal muscle polyglucosan bodies (6, 35). Our proteomic data similarly reveal, in human lymphoblasts, an altered protein unfolding response and ubiquitination (Table 2; Figure 1, networks 2–5) and autophagy (Figure 1, network 3) as a major part of the pathogenic process in APBD and adds to the linkage between autophagy and the pathogenesis of other disorders of glycogen metabolism (38).

Lafora disease is a severe childhood- or adolescence-onset neurodegenerative disorder characterized by refractory epilepsy, cognitive deficits and death, typically within 10 years of onset of clinical presentation. Lafora disease is caused by loss-of-function mutations in the *EPM2A* gene, coding for a protein phosphatase (laforin), or of the *NHLRC1* gene, coding for an E3 ubiquitin ligase (malin). The absence of either protein results in poorly branched, hyperphosphorylated glycogen that precipitates and aggregates into Lafora bodies in neurons and other tissues (39, 40). These intracellular inclusions are a principal driver of the neurological phenotype in Lafora disease, with the laforin-malin complex serving to regulate glycogen chain length. Pathophysiological processes that subsequently result from a deficiency of malin or laforin include varied and variable abnormalities of ubiquitin-proteasomal function, dysregulation of autophagy, abnormalities of aspects of cellular stress responses such as the endoplasmic reticulum stress response, dysregulation of AMPK, dysregulation of various mitochondrial functions, and activation of apoptosis (39, 40). Insofar as the clinical phenotypes and brain histopathologies are markedly different between APBD and Lafora disease and there also are differences in the structures of their glycogens (21), there necessarily must be significant differences in their underlying pathophysiologic processes and caution is needed in comparing disease mechanisms between these diseases. Nevertheless, similar to many of the themes of pathogenesis of Lafora disease, the proteomic data reported here reveal striking differential expression between APBD subjects and controls of many proteins, pathways and protein–protein networks related to the unfolded protein response, endoplasmic reticulum stress pathway, ubiquitinylation, mTOR signaling, and apoptosis.

A novel and unanticipated finding of this analysis relates to differences in RNA metabolism, especially translation, reflected in the highly differentially expressed EIF2, EIF4 and p70S6K and tRNA charging canonical pathways and in the protein–protein interaction network and upstream regulator analyses that also demonstrate these findings (Table 2; Figure 1 networks 1–3, 5; Supplementary Table 2). To our knowledge, prior APBD and Lafora disease investigations have not revealed these themes. Dysregulation of translation is a major contributing factor to the pathogenesis of multiple neurodegenerative disorders (41, 42). Its relevance to the pathogenesis of APBD and, possibly, Lafora disease, merits exploration.

The results of this study suggest several potentially informative additional areas of investigation including proteomic analyses of diverse non-lymphoblast tissues from persons and animal models with APBD, the investigation of lymphoblast proteomes of persons with different clinical subtypes of GSD IV, proteomic analyses of Lafora disease tissues, and the relevance of dysregulation of RNA translation. The proteomic approach described here has another potential usefulness. There are ongoing efforts – and some early successes – to screen for compounds of possible medical utility in the treatment of APBD (30, 33, 43). These largely rely on high throughput assays that evaluate impacts on the levels of polyglucosan bodies in model cell culture systems with or without drug exposure. Use of a lymphoblast proteomic assay as reported here might serve as an adjunctive follow-up approach to determine if promising pharmacologic candidates normalize the highly abnormal proteome of APBD lymphoblasts.

This study has several strengths. It supports and extends earlier histological data that showed polyglucosan accumulation in APBD-affected human lymphocytes. It does so by confirming several recently recognized pathophysiological processes and suggests several new disease-related processes in APBD, and in so doing establishes the lymphoblast system used here as another model system that can be applied in the *in vitro* study of APBD. As such, it can be used in the further analysis of pathways suggested by this work that have heretofore not been associated with the pathogenesis of APBD. Proteomic analyses of mutant lymphoblasts also has potential applicability in the assessment of possible medication therapy for APBD. Notwithstanding the attributes of this system, there are also pertinent limitations. The cellular physiology of lymphoblasts cannot perfectly model that of non-lymphoblast cell types, even when the involved pathways are expressed in lymphoblasts. Second, our bioinformatic analyses included only proteins that were quantified based on having at least two uniquely identifying peptides; we did not include proteins in those analyses whose levels would be based on only a single unique peptide. The approach used here therefore assures more robust protein measurements although we may be excluding some potentially significant proteins. Third, proteomic analysis by LC–MS/MS has analytic limitations. The form of LC–MS/MS used here did not allow for measurement of some biologically important protein modifications such as protein phosphorylation. In addition, proteins having extremely low levels of expression may not be detected and this, in turn, could result in absent detection of an important protein. Each of these analytic limitations can be mitigated; the former through use of LC–MS/MS specifically targeted to detect phosphorylated or selected other modifications of peptides and the latter by extension of the duration of the chromatographic separation prior to MS analysis or through fractionation of the cellular homogenate at the pre-MS stage and use of multiple LC–MS/MS analyses of each sample.

Finally, an intriguing additional result emerged from this study. This work on APBD is our third study using a LC–MS/MS proteomic analysis of lymphoblasts of a rare genetic CNS disorder; it was preceded by analyses of lymphoblast proteomes of persons with neurologic diseases due to mutations of *ARID1B* and *HERC2* (44, 45). In all three instances, proteomic analyses of an easily obtainable tissue – transformed peripheral blood lymphocytes – provided insights relevant to aspects of brain biology, presumably because of partial conservation of central biochemical processes even across highly differentiated tissues. This, in turn, raises the possibility that this approach may have a more generalizable utility; proteomic analyses of lymphoblasts may be particularly useful in investigations of the pathogenesis of uncommon or rare conditions for which human brain tissue is difficult to obtain.

## Data availability statement

The datasets presented in this study can be found in online repositories. The name of the repository and accession number can be found in the Materials and methods section.

## Ethics statement

The studies involving humans were approved by Cleveland Clinic Institutional Review Board. The studies were conducted in accordance with the local legislation and institutional requirements. The participants provided their written informed consent to participate in this study.

## Author contributions

JA: Formal analysis, Methodology, Writing – original draft, Writing – review & editing. FA: Formal analysis, Writing – review & editing. JB: Formal analysis, Writing – review & editing. DS: Methodology, Writing – review & editing. MN: Conceptualization, Formal analysis, Funding acquisition, Project administration, Supervision, Writing – original draft, Writing – review & editing.

## Footnotes

^1^www.qiagen.com/ingenuity
