# Supplementary material for: Proteomic investigations of adult polyglucosan body disease: insights into the pathobiology of a neurodegenerative disorder
Source: Front Neurol. 2023 Nov 14;14:1261125. doi: 10.3389/fneur.2023.1261125 (PMC10683643; doi:10.3389/fneur.2023.1261125)
Supplement: Supplementary file 1 [file Table_1.DOCX]

Supplementary Table 1. List of all differentially expressed proteins between APBD subjects and controls.

| Symbol | Uniprot Accession ID | Expr Log Ratio | p-value | False Discovery Rate (adjusted p-value) |
| --- | --- | --- | --- | --- |
| GBE1 | Q04446 | -3.43 | 2.50E-43 | 5.87E-40 |
| RRBP1 | Q9P2E9 | 0.90 | 3.63E-30 | 4.27E-27 |
| SPTBN1 | Q01082 | -0.34 | 6.23E-23 | 4.88E-20 |
| ALDOC | P09972 | -0.69 | 5.67E-20 | 3.33E-17 |
| SPTAN1 | Q13813 | -0.24 | 1.49E-19 | 7.00E-17 |
| RANGAP1 | P46060 | 0.51 | 5.67E-18 | 2.22E-15 |
| UGDH | O60701 | 0.80 | 1.31E-16 | 4.42E-14 |
| EVL | Q9UI08 | -0.64 | 1.56E-16 | 4.57E-14 |
| TLN1 | Q9Y490 | 0.14 | 2.91E-16 | 7.60E-14 |
| IPO5 | O00410 | 0.24 | 1.34E-15 | 3.15E-13 |
| ADA | P00813 | -0.62 | 2.57E-15 | 5.48E-13 |
| SND1 | Q7KZF4 | 0.23 | 2.92E-13 | 5.73E-11 |
| VIM | P08670 | 0.95 | 1.01E-12 | 1.83E-10 |
| MARCKSL1 | P49006 | -1.19 | 1.29E-12 | 2.17E-10 |
| FDPS | P14324 | -0.49 | 1.99E-12 | 3.12E-10 |
| NAMPT | P43490 | -0.42 | 2.48E-12 | 3.64E-10 |
| TPP2 | P29144 | 0.27 | 6.31E-12 | 8.72E-10 |
| NFKB2 | Q00653 | -0.60 | 1.10E-11 | 1.44E-09 |
| MTDH | Q86UE4 | 0.31 | 1.18E-11 | 1.46E-09 |
| IGHM | P01871 | 1.22 | 2.63E-11 | 3.09E-09 |
| GCN1 | Q92616 | 0.17 | 7.86E-11 | 8.80E-09 |
| HYOU1 | Q9Y4L1 | 0.41 | 1.17E-10 | 1.23E-08 |
| RFTN1 | Q14699 | -0.43 | 1.21E-10 | 1.23E-08 |
| HSP90B1 | P14625 | 0.33 | 1.38E-10 | 1.35E-08 |
| RRM1 | P23921 | 0.33 | 1.67E-10 | 1.57E-08 |
| PCNA | P12004 | 0.48 | 2.86E-10 | 2.58E-08 |
| MCM2 | P49736 | 0.23 | 4.52E-10 | 3.94E-08 |
| FSCN1 | Q16658 | -0.49 | 4.80E-10 | 4.03E-08 |
| MCM5 | P33992 | 0.28 | 2.19E-09 | 1.71E-07 |
| VASP | P50552 | -0.34 | 2.11E-09 | 1.71E-07 |
| COPG1 | Q9Y678 | 0.18 | 2.69E-09 | 2.04E-07 |
| PRMT1 | Q99873 | 0.47 | 2.88E-09 | 2.12E-07 |
| MCM3 | P25205 | 0.22 | 4.07E-09 | 2.90E-07 |
| CSDE1 | O75534 | 0.35 | 6.84E-09 | 4.73E-07 |
| FAH | P16930 | 0.60 | 7.93E-09 | 5.32E-07 |
| EEA1 | Q15075 | -0.41 | 9.27E-09 | 5.89E-07 |
| FTO | Q9C0B1 | 0.43 | 9.25E-09 | 5.89E-07 |
| HSPA5 | P11021 | 0.31 | 1.25E-08 | 7.72E-07 |
| RARS | P54136 | 0.18 | 1.39E-08 | 8.40E-07 |
| QARS | P47897 | 0.24 | 1.47E-08 | 8.62E-07 |
| DDX5 | P17844 | 0.33 | 1.63E-08 | 9.36E-07 |
| EEF1G | P26641 | 0.30 | 3.89E-08 | 2.18E-06 |
| GRPEL1 | Q9HAV7 | -0.32 | 4.46E-08 | 2.44E-06 |
| CASP3 | P42574 | 0.60 | 4.61E-08 | 2.46E-06 |
| LAP3 | P28838 | 0.20 | 7.11E-08 | 3.72E-06 |
| SQSTM1 | Q13501 | -0.68 | 8.56E-08 | 4.38E-06 |
| AP2B1 | P63010 | 0.25 | 8.99E-08 | 4.40E-06 |
| HARS | P12081 | 0.20 | 8.93E-08 | 4.40E-06 |
| GATM | P50440 | -0.63 | 9.35E-08 | 4.49E-06 |
| SEC24C | P53992 | 0.15 | 1.02E-07 | 4.79E-06 |
| PSIP1 | O75475 | -0.36 | 1.22E-07 | 5.63E-06 |
| NAPA | P54920 | 0.19 | 1.57E-07 | 7.10E-06 |
| PFKP | Q01813 | -0.16 | 1.74E-07 | 7.74E-06 |
| NPEPPS | P55786 | 0.23 | 2.35E-07 | 1.02E-05 |
| PPP2CA | P67775 | 0.19 | 3.00E-07 | 1.28E-05 |
| LMNB2 | Q03252 | -0.17 | 3.26E-07 | 1.37E-05 |
| CYB5R3 | P00387 | -0.34 | 4.54E-07 | 1.79E-05 |
| EPS15 | P42566 | -0.31 | 4.57E-07 | 1.79E-05 |
| IQGAP1 | P46940 | 0.11 | 4.54E-07 | 1.79E-05 |
| TARS | P26639 | 0.13 | 4.41E-07 | 1.79E-05 |
| PLG | P00747 | -0.92 | 4.68E-07 | 1.80E-05 |
| STAT2 | P52630 | 0.34 | 6.24E-07 | 2.37E-05 |
| MCM4 | P33991 | 0.24 | 6.61E-07 | 2.47E-05 |
| CDV3 | Q9UKY7 | 0.41 | 7.77E-07 | 2.85E-05 |
| RPS7 | P62081 | 0.25 | 9.31E-07 | 3.37E-05 |
| DYNC1H1 | Q14204 | 0.10 | 9.63E-07 | 3.43E-05 |
| DUT | P33316 | 0.45 | 1.05E-06 | 3.69E-05 |
| PCK2 | Q16822 | -0.26 | 1.40E-06 | 4.83E-05 |
| NCAPD2 | Q15021 | 0.26 | 1.44E-06 | 4.85E-05 |
| PPP1R9B | Q96SB3 | -0.38 | 1.43E-06 | 4.85E-05 |
| HM13 | Q8TCT9 | 0.38 | 1.58E-06 | 5.16E-05 |
| HSPA4L | O95757 | -0.42 | 1.57E-06 | 5.16E-05 |
| LRMP | Q12912 | -0.90 | 1.83E-06 | 5.91E-05 |
| LRPPRC | P42704 | 0.13 | 1.94E-06 | 6.17E-05 |
| IGLL1/IGLL5 | B9A064 | 1.48 | 2.03E-06 | 6.35E-05 |
| MKI67 | P46013 | 0.21 | 2.09E-06 | 6.45E-05 |
| DPYSL2 | Q16555 | -0.56 | 2.62E-06 | 7.99E-05 |
| HSP90AB1 | P08238 | -0.16 | 2.69E-06 | 8.12E-05 |
| PGK1 | P00558 | -0.23 | 3.02E-06 | 8.98E-05 |
| ICAM1 | P05362 | -0.80 | 3.16E-06 | 9.18E-05 |
| PSME2 | Q9UL46 | 0.22 | 3.16E-06 | 9.18E-05 |
| ITGB7 | P26010 | 1.13 | 3.41E-06 | 9.78E-05 |
| KPNA2 | P52292 | 0.67 | 4.27E-06 | 1.21E-04 |
| ALB | P02768 | -1.15 | 4.52E-06 | 1.26E-04 |
| DOK3 | Q7L591 | -0.59 | 5.00E-06 | 1.38E-04 |
| VCP | P55072 | 0.17 | 5.43E-06 | 1.49E-04 |
| RPS25 | P62851 | 0.21 | 5.68E-06 | 1.52E-04 |
| TRIM25 | Q14258 | 0.20 | 5.68E-06 | 1.52E-04 |
| FARSB | Q9NSD9 | 0.16 | 5.86E-06 | 1.53E-04 |
| UBE2O | Q9C0C9 | 0.41 | 5.86E-06 | 1.53E-04 |
| MS4A1 | P11836 | -0.43 | 6.67E-06 | 1.72E-04 |
| ZC3HAV1 | Q7Z2W4 | 0.37 | 8.00E-06 | 2.05E-04 |
| AIP | O00170 | -0.25 | 8.69E-06 | 2.20E-04 |
| UPF1 | Q92900 | 0.16 | 9.29E-06 | 2.32E-04 |
| FKBP11 | Q9NYL4 | 0.59 | 1.22E-05 | 2.94E-04 |
| PSMD2 | Q13200 | 0.17 | 1.21E-05 | 2.94E-04 |
| SRPRB | Q9Y5M8 | 0.31 | 1.23E-05 | 2.94E-04 |
| TXNDC5 | Q8NBS9 | 0.57 | 1.21E-05 | 2.94E-04 |
| USP15 | Q9Y4E8 | 0.20 | 1.36E-05 | 3.22E-04 |
| ENO2 | P09104 | -0.44 | 1.61E-05 | 3.71E-04 |
| FNBP1 | Q96RU3 | -0.20 | 1.60E-05 | 3.71E-04 |
| MCM6 | Q14566 | 0.20 | 1.59E-05 | 3.71E-04 |
| DNMT1 | P26358 | 0.31 | 1.65E-05 | 3.76E-04 |
| RPS8 | P62241 | 0.19 | 1.78E-05 | 4.03E-04 |
| USP5 | P45974 | 0.14 | 1.87E-05 | 4.18E-04 |
| RPS3 | P23396 | 0.17 | 1.91E-05 | 4.24E-04 |
| PSME3 | P61289 | 0.32 | 1.99E-05 | 4.37E-04 |
| DNAJC7 | Q99615 | 0.29 | 2.15E-05 | 4.65E-04 |
| RPS13 | P62277 | 0.21 | 2.16E-05 | 4.65E-04 |
| ANXA5 | P08758 | 0.24 | 2.24E-05 | 4.77E-04 |
| SRP54 | P61011 | 0.18 | 2.25E-05 | 4.77E-04 |
| P4HB | P07237 | 0.22 | 2.36E-05 | 4.96E-04 |
| API5 | Q9BZZ5 | 0.16 | 2.47E-05 | 5.10E-04 |
| NAGK | Q9UJ70 | 0.48 | 2.50E-05 | 5.10E-04 |
| THEMIS2 | Q5TEJ8 | -0.64 | 2.48E-05 | 5.10E-04 |
| NME2 | P22392 | 0.41 | 2.70E-05 | 5.48E-04 |
| RSL1D1 | O76021 | -0.16 | 2.84E-05 | 5.72E-04 |
| MSMO1 | Q15800 | -0.53 | 2.95E-05 | 5.87E-04 |
| FUS | P35637 | 0.18 | 2.98E-05 | 5.88E-04 |
| MCM7 | P33993 | 0.23 | 3.03E-05 | 5.94E-04 |
| ATP2A2 | P16615 | 0.15 | 3.26E-05 | 6.33E-04 |
| CIP2A | Q8TCG1 | 0.61 | 3.41E-05 | 6.57E-04 |
| FASN | P49327 | 0.14 | 3.52E-05 | 6.73E-04 |
| AP2A1 | O95782 | 0.17 | 3.68E-05 | 6.98E-04 |
| ILF3 | Q12906 | 0.12 | 3.85E-05 | 7.23E-04 |
| SEL1L | Q9UBV2 | 0.60 | 3.97E-05 | 7.41E-04 |
| DCPS | Q96C86 | -0.44 | 4.09E-05 | 7.57E-04 |
| NCF1B | A6NI72 | -0.44 | 4.13E-05 | 7.57E-04 |
| NUP210 | Q8TEM1 | -0.30 | 4.15E-05 | 7.57E-04 |
| ATP1B3 | P54709 | 0.33 | 4.23E-05 | 7.66E-04 |
| ISG20 | Q96AZ6 | 0.41 | 4.65E-05 | 8.34E-04 |
| CAND1 | Q86VP6 | 0.15 | 4.76E-05 | 8.49E-04 |
| PNP | P00491 | 0.41 | 4.86E-05 | 8.58E-04 |
| TXNL1 | O43396 | 0.22 | 5.06E-05 | 8.87E-04 |
| DNAJC9 | Q8WXX5 | 0.23 | 5.55E-05 | 9.66E-04 |
| DDX3X | O00571 | 0.19 | 5.62E-05 | 9.71E-04 |
| RACK1 | P63244 | 0.16 | 5.70E-05 | 9.79E-04 |
| CDK1 | P06493 | 0.35 | 5.85E-05 | 9.87E-04 |
| CYCS | P99999 | 0.36 | 5.87E-05 | 9.87E-04 |
| EIF4G1 | Q04637 | 0.18 | 5.87E-05 | 9.87E-04 |
| NUP160 | Q12769 | 0.21 | 6.00E-05 | 1.00E-03 |
| COPS7A | Q9UBW8 | 0.56 | 6.29E-05 | 1.03E-03 |
| PSPC1 | Q8WXF1 | -0.21 | 6.28E-05 | 1.03E-03 |
| EIF3B | P55884 | 0.12 | 6.62E-05 | 1.07E-03 |
| RRM2 | P31350 | 0.56 | 6.63E-05 | 1.07E-03 |
| ANXA2 | P07355 | 0.19 | 6.78E-05 | 1.08E-03 |
| CDC5L | Q99459 | 0.16 | 6.69E-05 | 1.08E-03 |
| COPB1 | P53618 | 0.17 | 7.42E-05 | 1.18E-03 |
| PRDX3 | P30048 | -0.16 | 7.94E-05 | 1.25E-03 |
| COMMD4 | Q9H0A8 | 0.33 | 8.14E-05 | 1.27E-03 |
| SH3GL1 | Q99961 | -0.28 | 8.16E-05 | 1.27E-03 |
| DARS | P14868 | -0.11 | 8.34E-05 | 1.29E-03 |
| SUPT5H | O00267 | 0.27 | 8.48E-05 | 1.30E-03 |
| INPP5D | Q92835 | -0.27 | 8.66E-05 | 1.32E-03 |
| C1QBP | Q07021 | -0.25 | 9.11E-05 | 1.36E-03 |
| CD82 | P27701 | -0.41 | 9.00E-05 | 1.36E-03 |
| SMC4 | Q9NTJ3 | 0.21 | 9.00E-05 | 1.36E-03 |
| HIBCH | Q6NVY1 | 0.36 | 9.19E-05 | 1.37E-03 |
| PABPC1 | P11940 | 0.25 | 9.45E-05 | 1.40E-03 |
| LY75 | O60449 | -0.46 | 9.64E-05 | 1.42E-03 |
| ERAP1 | Q9NZ08 | 0.42 | 1.03E-04 | 1.51E-03 |
| DDB1 | Q16531 | 0.12 | 1.06E-04 | 1.53E-03 |
| RBM3 | P98179 | 1.78 | 1.10E-04 | 1.59E-03 |
| SRP68 | Q9UHB9 | 0.17 | 1.18E-04 | 1.69E-03 |
| UBE2G1 | P62253 | 0.45 | 1.23E-04 | 1.75E-03 |
| YWHAG | P61981 | -0.17 | 1.26E-04 | 1.78E-03 |
| MATR3 | P43243 | 0.10 | 1.37E-04 | 1.94E-03 |
| MACF1 | Q9UPN3 | -0.27 | 1.44E-04 | 1.99E-03 |
| NOP58 | Q9Y2X3 | 0.15 | 1.44E-04 | 1.99E-03 |
| UGGT1 | Q9NYU2 | 0.17 | 1.44E-04 | 1.99E-03 |
| ADSL | P30566 | 0.22 | 1.45E-04 | 2.00E-03 |
| AP3B1 | O00203 | 0.22 | 1.46E-04 | 2.00E-03 |
| COX4I1 | P13073 | 0.22 | 1.47E-04 | 2.00E-03 |
| POLR2B | P30876 | 0.24 | 1.48E-04 | 2.00E-03 |
| NANS | Q9NR45 | 0.28 | 1.51E-04 | 2.03E-03 |
| RPS2 | P15880 | 0.25 | 1.57E-04 | 2.09E-03 |
| RPS14 | P62263 | 0.24 | 1.58E-04 | 2.10E-03 |
| ETF1 | P62495 | 0.17 | 1.62E-04 | 2.14E-03 |
| ACO2 | Q99798 | -0.15 | 1.67E-04 | 2.17E-03 |
| CLUH | O75153 | 0.32 | 1.68E-04 | 2.17E-03 |
| SUPT6H | Q7KZ85 | 0.33 | 1.65E-04 | 2.17E-03 |
| UROD | P06132 | 0.37 | 1.67E-04 | 2.17E-03 |
| RPL12 | P30050 | 0.17 | 1.71E-04 | 2.19E-03 |
| PSMA4 | P25789 | 0.11 | 1.72E-04 | 2.20E-03 |
| ELAC2 | Q9BQ52 | 0.33 | 1.74E-04 | 2.21E-03 |
| BAZ1B | Q9UIG0 | 0.28 | 1.78E-04 | 2.24E-03 |
| HNRNPL | P14866 | -0.14 | 1.78E-04 | 2.24E-03 |
| MRPL1 | Q9BYD6 | 0.20 | 1.81E-04 | 2.26E-03 |
| STX6 | O43752 | -0.47 | 1.83E-04 | 2.27E-03 |
| GLS | O94925 | 0.20 | 1.88E-04 | 2.31E-03 |
| PDIA6 | Q15084 | 0.25 | 1.87E-04 | 2.31E-03 |
| PLIN3 | O60664 | -0.22 | 1.90E-04 | 2.31E-03 |
| RCSD1 | Q6JBY9 | -0.33 | 1.90E-04 | 2.31E-03 |
| PFAS | O15067 | 0.20 | 1.93E-04 | 2.33E-03 |
| SEC13 | P55735 | 0.17 | 2.01E-04 | 2.42E-03 |
| MFN1 | Q8IWA4 | -0.70 | 2.04E-04 | 2.44E-03 |
| P4HA1 | P13674 | -0.23 | 2.06E-04 | 2.46E-03 |
| LCP1 | P13796 | 0.12 | 2.09E-04 | 2.48E-03 |
| PUF60 | Q9UHX1 | 0.14 | 2.19E-04 | 2.58E-03 |
| RPS21 | P63220 | 0.28 | 2.27E-04 | 2.67E-03 |
| EIF4G2 | P78344 | 0.17 | 2.33E-04 | 2.72E-03 |
| RPL15 | P61313 | 0.20 | 2.34E-04 | 2.72E-03 |
| EIF2A | Q9BY44 | 0.24 | 2.37E-04 | 2.75E-03 |
| COASY | Q13057 | 0.39 | 2.45E-04 | 2.81E-03 |
| CTSD | P07339 | 0.43 | 2.46E-04 | 2.81E-03 |
| EVI2B | P34910 | 0.77 | 2.45E-04 | 2.81E-03 |
| PFKL | P17858 | -0.17 | 2.48E-04 | 2.82E-03 |
| RTRAF | Q9Y224 | 0.22 | 2.49E-04 | 2.82E-03 |
| MANF | P55145 | 0.38 | 2.51E-04 | 2.83E-03 |
| CSNK2A2 | P19784 | 0.28 | 2.58E-04 | 2.88E-03 |
| TCEA1 | P23193 | -0.13 | 2.58E-04 | 2.88E-03 |
| ITGA4 | P13612 | 0.48 | 2.64E-04 | 2.93E-03 |
| U2SURP | O15042 | 0.18 | 2.72E-04 | 3.00E-03 |
| SRGN | P10124 | -0.77 | 2.75E-04 | 3.01E-03 |
| TRAF3IP3 | Q9Y228 | 0.90 | 2.75E-04 | 3.01E-03 |
| RBM8A | Q9Y5S9 | 0.17 | 2.80E-04 | 3.05E-03 |
| RPS4X | P62701 | 0.17 | 2.85E-04 | 3.09E-03 |
| ERO1A | Q96HE7 | -0.17 | 2.93E-04 | 3.16E-03 |
| SLC16A3 | O15427 | -0.73 | 2.97E-04 | 3.18E-03 |
| EHD4 | Q9H223 | -0.19 | 2.99E-04 | 3.20E-03 |
| EIF3E | P60228 | 0.11 | 3.08E-04 | 3.28E-03 |
| SART3 | Q15020 | -0.17 | 3.20E-04 | 3.39E-03 |
| HNRNPM | P52272 | 0.09 | 3.26E-04 | 3.42E-03 |
| SMC2 | O95347 | 0.19 | 3.25E-04 | 3.42E-03 |
| FAM162A | Q96A26 | -0.27 | 3.28E-04 | 3.43E-03 |
| FAM3C | Q92520 | -0.45 | 3.42E-04 | 3.52E-03 |
| PRRC2C | Q9Y520 | 0.27 | 3.39E-04 | 3.52E-03 |
| RCN1 | Q15293 | -0.31 | 3.41E-04 | 3.52E-03 |
| TMEM214 | Q6NUQ4 | 0.39 | 3.52E-04 | 3.61E-03 |
| ARL1 | P40616 | 0.25 | 3.71E-04 | 3.76E-03 |
| CPSF2 | Q9P2I0 | 0.23 | 3.70E-04 | 3.76E-03 |
| PRDX1 | Q06830 | -0.14 | 3.71E-04 | 3.76E-03 |
| ANXA4 | P09525 | -0.25 | 3.74E-04 | 3.77E-03 |
| PIN1 | Q13526 | 0.38 | 3.76E-04 | 3.78E-03 |
| PPP2R2A | P63151 | 0.28 | 3.84E-04 | 3.84E-03 |
| VPS25 | Q9BRG1 | 0.48 | 3.85E-04 | 3.84E-03 |
| COX6B1 | P14854 | 0.34 | 3.93E-04 | 3.90E-03 |
| MRE11 | P49959 | -0.20 | 3.98E-04 | 3.93E-03 |
| ARCN1 | P48444 | 0.13 | 4.02E-04 | 3.95E-03 |
| PDCD11 | Q14690 | 0.14 | 4.11E-04 | 4.02E-03 |
| BUB3 | O43684 | 0.17 | 4.17E-04 | 4.07E-03 |
| PRIM2 | P49643 | 0.55 | 4.21E-04 | 4.09E-03 |
| DDX24 | Q9GZR7 | -0.21 | 4.28E-04 | 4.14E-03 |
| PTPN1 | P18031 | -0.23 | 4.30E-04 | 4.14E-03 |
| MSH2 | P43246 | 0.19 | 4.46E-04 | 4.28E-03 |
| NDUFS1 | P28331 | 0.16 | 4.62E-04 | 4.41E-03 |
| HSPD1 | P10809 | -0.10 | 4.68E-04 | 4.46E-03 |
| GLUD1 | P00367 | -0.16 | 4.72E-04 | 4.47E-03 |
| RHOG | P84095 | -0.38 | 4.78E-04 | 4.51E-03 |
| RPS5 | P46782 | 0.19 | 4.89E-04 | 4.58E-03 |
| TPI1 | P60174 | -0.15 | 4.88E-04 | 4.58E-03 |
| HPCA | P84074 | -0.98 | 5.01E-04 | 4.68E-03 |
| CELF2 | O95319 | 0.23 | 5.08E-04 | 4.72E-03 |
| EIF4A1 | P60842 | 0.21 | 5.19E-04 | 4.80E-03 |
| MZB1 | Q8WU39 | 0.49 | 5.25E-04 | 4.82E-03 |
| RPS18 | P62269 | 0.17 | 5.27E-04 | 4.82E-03 |
| TPT1 | P13693 | 0.28 | 5.24E-04 | 4.82E-03 |
| HNRNPA0 | Q13151 | 0.23 | 5.40E-04 | 4.92E-03 |
| GLG1 | Q92896 | 0.26 | 5.51E-04 | 5.00E-03 |
| UNC13D | Q70J99 | -0.48 | 5.56E-04 | 5.03E-03 |
| FKBP4 | Q02790 | -0.14 | 5.61E-04 | 5.05E-03 |
| MAPRE1 | Q15691 | 0.20 | 5.81E-04 | 5.22E-03 |
| DCTN1 | Q14203 | 0.17 | 5.92E-04 | 5.29E-03 |
| ADSS | P30520 | 0.18 | 6.01E-04 | 5.35E-03 |
| IRF4 | Q15306 | 0.19 | 6.04E-04 | 5.35E-03 |
| PDHB | P11177 | -0.22 | 6.07E-04 | 5.37E-03 |
| PSMA7 | O14818 | 0.13 | 6.10E-04 | 5.37E-03 |
| CORO1C | Q9ULV4 | -0.17 | 6.16E-04 | 5.40E-03 |
| PSMD9 | O00233 | 0.29 | 6.20E-04 | 5.42E-03 |
| EEF2 | P13639 | 0.18 | 6.35E-04 | 5.51E-03 |
| KPNB1 | Q14974 | 0.14 | 6.33E-04 | 5.51E-03 |
| FABP5 | Q01469 | 0.47 | 6.88E-04 | 5.95E-03 |
| RPL4 | P36578 | 0.18 | 7.00E-04 | 6.03E-03 |
| IFI30 | P13284 | -0.44 | 7.06E-04 | 6.06E-03 |
| CNDP2 | Q96KP4 | -0.14 | 7.19E-04 | 6.15E-03 |
| RPL36 | Q9Y3U8 | 0.21 | 7.37E-04 | 6.28E-03 |
| ALDH16A1 | Q8IZ83 | 0.39 | 7.59E-04 | 6.43E-03 |
| GLMN | Q92990 | 0.35 | 7.72E-04 | 6.43E-03 |
| HUWE1 | Q7Z6Z7 | -0.12 | 7.61E-04 | 6.43E-03 |
| PPP2R1A | P30153 | 0.13 | 7.70E-04 | 6.43E-03 |
| SLC25A1 | P53007 | -0.28 | 7.68E-04 | 6.43E-03 |
| TPM4 | P67936 | -0.24 | 7.67E-04 | 6.43E-03 |
| AP3D1 | O14617 | 0.19 | 7.82E-04 | 6.50E-03 |
| PDCD4 | Q53EL6 | -0.26 | 7.94E-04 | 6.57E-03 |
| CCDC47 | Q96A33 | 0.24 | 8.04E-04 | 6.63E-03 |
| AHSG | P02765 | -0.68 | 8.20E-04 | 6.74E-03 |
| PPFIBP2 | Q8ND30 | 0.57 | 8.44E-04 | 6.92E-03 |
| ARMCX3 | Q9UH62 | 0.51 | 8.52E-04 | 6.96E-03 |
| STK10 | O94804 | -0.22 | 8.66E-04 | 7.04E-03 |
| RPL27 | P61353 | 0.18 | 8.74E-04 | 7.08E-03 |
| RPL32 | P62910 | 0.24 | 8.77E-04 | 7.08E-03 |
| PSMC1 | P62191 | 0.16 | 8.97E-04 | 7.22E-03 |
| LMAN2 | Q12907 | 0.50 | 9.13E-04 | 7.32E-03 |
| SIT1 | Q9Y3P8 | 0.61 | 9.21E-04 | 7.36E-03 |
| EEF1A1 | P68104 | 0.21 | 9.34E-04 | 7.39E-03 |
| WDFY1 | Q8IWB7 | -0.38 | 9.32E-04 | 7.39E-03 |
| YY1 | P25490 | -0.40 | 9.32E-04 | 7.39E-03 |
| LPXN | O60711 | -0.35 | 9.49E-04 | 7.49E-03 |
| TECR | Q9NZ01 | 0.29 | 9.77E-04 | 7.66E-03 |
| CPT2 | P23786 | -0.58 | 9.89E-04 | 7.73E-03 |
| HSPB11 | Q9Y547 | 0.44 | 1.04E-03 | 8.11E-03 |
| SRPRA | P08240 | 0.27 | 1.07E-03 | 8.28E-03 |
| CAB39 | Q9Y376 | 0.20 | 1.08E-03 | 8.38E-03 |
| YBX1 | P67809 | 0.18 | 1.11E-03 | 8.57E-03 |
| HSPA9 | P38646 | -0.08 | 1.19E-03 | 9.15E-03 |
| UCHL5 | Q9Y5K5 | 0.18 | 1.19E-03 | 9.15E-03 |
| RAB9A | P51151 | -0.29 | 1.20E-03 | 9.18E-03 |
| EIF1 | P41567 | 0.67 | 1.22E-03 | 9.29E-03 |
| HNRNPUL2 | Q1KMD3 | -0.13 | 1.24E-03 | 9.42E-03 |
| STRAP | Q9Y3F4 | 0.20 | 1.28E-03 | 9.65E-03 |
| IDE | P14735 | 0.25 | 1.29E-03 | 9.72E-03 |
| ACSL4 | O60488 | -0.25 | 1.31E-03 | 9.86E-03 |
| BCKDK | O14874 | -0.57 | 1.36E-03 | 1.02E-02 |
| TRAPPC3 | O43617 | 0.24 | 1.37E-03 | 1.02E-02 |
| RBMX | P38159 | 0.28 | 1.42E-03 | 1.05E-02 |
| HK2 | P52789 | 0.16 | 1.43E-03 | 1.06E-02 |
| ESYT1 | Q9BSJ8 | 0.25 | 1.47E-03 | 1.09E-02 |
| PRKRA | O75569 | -0.35 | 1.49E-03 | 1.10E-02 |
| RAB11A | P62491 | -0.16 | 1.54E-03 | 1.13E-02 |
| DENR | O43583 | 0.23 | 1.56E-03 | 1.14E-02 |
| CKB | P12277 | -0.94 | 1.59E-03 | 1.16E-02 |
| AAAS | Q9NRG9 | 0.32 | 1.61E-03 | 1.17E-02 |
| COPS8 | Q99627 | 0.15 | 1.62E-03 | 1.17E-02 |
| PEPD | P12955 | 0.25 | 1.61E-03 | 1.17E-02 |
| ELOC | Q15369 | 0.15 | 1.65E-03 | 1.19E-02 |
| ACTR3 | P61158 | 0.11 | 1.67E-03 | 1.20E-02 |
| MCTS1 | Q9ULC4 | 0.17 | 1.73E-03 | 1.24E-02 |
| COPS4 | Q9BT78 | 0.15 | 1.76E-03 | 1.25E-02 |
| DTYMK | P23919 | 0.18 | 1.74E-03 | 1.25E-02 |
| COPA | P53621 | 0.10 | 1.77E-03 | 1.26E-02 |
| HDGF | P51858 | -0.13 | 1.78E-03 | 1.26E-02 |
| EIF3A | Q14152 | 0.08 | 1.83E-03 | 1.29E-02 |
| OAS2 | P29728 | -0.17 | 1.85E-03 | 1.30E-02 |
| PSME1 | Q06323 | 0.11 | 1.85E-03 | 1.30E-02 |
| MRTO4 | Q9UKD2 | 0.26 | 1.87E-03 | 1.31E-02 |
| ALYREF | Q86V81 | 0.19 | 1.95E-03 | 1.36E-02 |
| CDS2 | O95674 | -0.40 | 1.97E-03 | 1.36E-02 |
| CNN2 | Q99439 | 0.25 | 1.97E-03 | 1.36E-02 |
| NFKB1 | P19838 | -0.18 | 1.97E-03 | 1.36E-02 |
| SRM | P19623 | 0.17 | 1.97E-03 | 1.36E-02 |
| EDC4 | Q6P2E9 | 0.16 | 2.00E-03 | 1.38E-02 |
| MYO18A | Q92614 | 0.19 | 2.13E-03 | 1.46E-02 |
| STX18 | Q9P2W9 | 0.36 | 2.13E-03 | 1.46E-02 |
| THOC1 | Q96FV9 | 0.19 | 2.17E-03 | 1.48E-02 |
| DDOST | P39656 | 0.15 | 2.20E-03 | 1.49E-02 |
| NNT | Q13423 | -0.13 | 2.19E-03 | 1.49E-02 |
| NACC1 | Q96RE7 | 0.36 | 2.22E-03 | 1.50E-02 |
| COPS3 | Q9UNS2 | 0.26 | 2.25E-03 | 1.51E-02 |
| CTSS | P25774 | 0.36 | 2.24E-03 | 1.51E-02 |
| GRSF1 | Q12849 | 0.37 | 2.26E-03 | 1.52E-02 |
| VTN | P04004 | -0.74 | 2.31E-03 | 1.54E-02 |
| CD44 | P16070 | 0.34 | 2.45E-03 | 1.62E-02 |
| RPL5 | P46777 | 0.12 | 2.44E-03 | 1.62E-02 |
| ANAPC7 | Q9UJX3 | 0.29 | 2.50E-03 | 1.65E-02 |
| DDX46 | Q7L014 | 0.13 | 2.51E-03 | 1.65E-02 |
| UBA5 | Q9GZZ9 | 0.23 | 2.55E-03 | 1.68E-02 |
| CCT5 | P48643 | 0.08 | 2.63E-03 | 1.73E-02 |
| CLTC | Q00610 | 0.07 | 2.65E-03 | 1.73E-02 |
| SEC31A | O94979 | 0.20 | 2.65E-03 | 1.73E-02 |
| CARM1 | Q86X55 | -0.25 | 2.72E-03 | 1.77E-02 |
| CBX3 | Q13185 | 0.12 | 2.85E-03 | 1.85E-02 |
| CMPK1 | P30085 | -0.21 | 2.86E-03 | 1.85E-02 |
| OGFR | Q9NZT2 | -0.20 | 2.87E-03 | 1.85E-02 |
| GMPS | P49915 | 0.12 | 2.99E-03 | 1.91E-02 |
| IPO4 | Q8TEX9 | 0.15 | 2.98E-03 | 1.91E-02 |
| PDIA3 | P30101 | 0.12 | 2.96E-03 | 1.91E-02 |
| TSN | Q15631 | 0.22 | 3.02E-03 | 1.92E-02 |
| TWF2 | Q6IBS0 | 0.28 | 3.01E-03 | 1.92E-02 |
| RPS11 | P62280 | 0.18 | 3.06E-03 | 1.94E-02 |
| DLAT | P10515 | -0.15 | 3.09E-03 | 1.96E-02 |
| DNAJA1 | P31689 | 0.18 | 3.14E-03 | 1.98E-02 |
| FBLL1 | A6NHQ2 | 0.17 | 3.13E-03 | 1.98E-02 |
| PKN1 | Q16512 | 0.25 | 3.16E-03 | 1.98E-02 |
| PSMA1 | P25786 | 0.13 | 3.14E-03 | 1.98E-02 |
| NRDC | O43847 | 0.77 | 3.18E-03 | 1.99E-02 |
| PPP1CC | P36873 | 0.21 | 3.19E-03 | 1.99E-02 |
| CAPRIN1 | Q14444 | 0.29 | 3.25E-03 | 2.02E-02 |
| PDIA4 | P13667 | 0.15 | 3.28E-03 | 2.03E-02 |
| GARS | P41250 | -0.16 | 3.42E-03 | 2.11E-02 |
| MTREX | P42285 | 0.14 | 3.46E-03 | 2.13E-02 |
| LONP1 | P36776 | -0.26 | 3.54E-03 | 2.17E-02 |
| PYCR1 | P32322 | -0.24 | 3.54E-03 | 2.17E-02 |
| COPB2 | P35606 | 0.12 | 3.58E-03 | 2.19E-02 |
| HIST1H2BA | Q96A08 | -0.30 | 3.60E-03 | 2.20E-02 |
| LSP1 | P33241 | 0.38 | 3.73E-03 | 2.27E-02 |
| BCAP31 | P51572 | 0.17 | 3.77E-03 | 2.28E-02 |
| DDX19A | Q9NUU7 | 0.13 | 3.75E-03 | 2.28E-02 |
| PPIB | P23284 | 0.34 | 3.76E-03 | 2.28E-02 |
| SRP72 | O76094 | 0.22 | 3.77E-03 | 2.28E-02 |
| PSMA2 | P25787 | 0.30 | 3.81E-03 | 2.29E-02 |
| NOL6 | Q9H6R4 | 0.20 | 3.86E-03 | 2.31E-02 |
| UTP14A | Q9BVJ6 | 0.20 | 3.85E-03 | 2.31E-02 |
| DRG1 | Q9Y295 | 0.12 | 3.94E-03 | 2.35E-02 |
| GGH | Q92820 | 0.25 | 3.98E-03 | 2.36E-02 |
| GFM1 | Q96RP9 | 0.15 | 4.03E-03 | 2.39E-02 |
| SASH3 | O75995 | -0.23 | 4.04E-03 | 2.39E-02 |
| CD40 | P25942 | -0.58 | 4.11E-03 | 2.41E-02 |
| NDUFA9 | Q16795 | 0.17 | 4.10E-03 | 2.41E-02 |
| NT5C3A | Q9H0P0 | 0.22 | 4.13E-03 | 2.42E-02 |
| DNAJC3 | Q13217 | 0.57 | 4.20E-03 | 2.43E-02 |
| SLC1A5 | Q15758 | 0.36 | 4.16E-03 | 2.43E-02 |
| SLC3A2 | P08195 | 0.16 | 4.20E-03 | 2.43E-02 |
| TTC37 | Q6PGP7 | -0.28 | 4.18E-03 | 2.43E-02 |
| UNC45A | Q9H3U1 | 0.35 | 4.16E-03 | 2.43E-02 |
| PDAP1 | Q13442 | 0.17 | 4.22E-03 | 2.44E-02 |
| SLC2A3 | P11169 | -0.57 | 4.31E-03 | 2.48E-02 |
| RPS23 | P62266 | 0.23 | 4.32E-03 | 2.49E-02 |
| SERBP1 | Q8NC51 | -0.29 | 4.40E-03 | 2.53E-02 |
| ASCC3 | Q8N3C0 | 0.22 | 4.54E-03 | 2.59E-02 |
| CSNK2A1 | P68400 | 0.13 | 4.54E-03 | 2.59E-02 |
| SRP14 | P37108 | 0.16 | 4.56E-03 | 2.60E-02 |
| SERPINB9 | P50453 | -0.32 | 4.65E-03 | 2.64E-02 |
| SFXN1 | Q9H9B4 | -0.17 | 4.68E-03 | 2.64E-02 |
| SMARCA5 | O60264 | 0.11 | 4.69E-03 | 2.64E-02 |
| UBAP2L | Q14157 | 0.13 | 4.66E-03 | 2.64E-02 |
| CUL4B | Q13620 | 0.23 | 4.71E-03 | 2.65E-02 |
| TIA1 | P31483 | 0.23 | 4.76E-03 | 2.67E-02 |
| THOC2 | Q8NI27 | 0.25 | 4.85E-03 | 2.71E-02 |
| CTSZ | Q9UBR2 | 0.85 | 4.87E-03 | 2.72E-02 |
| FAM129A | Q9BZQ8 | 0.37 | 4.90E-03 | 2.73E-02 |
| NCLN | Q969V3 | 0.27 | 4.92E-03 | 2.73E-02 |
| SSB | P05455 | 0.12 | 4.91E-03 | 2.73E-02 |
| RPS16 | P62249 | 0.14 | 5.01E-03 | 2.77E-02 |
| RNF20 | Q5VTR2 | -0.42 | 5.06E-03 | 2.79E-02 |
| COL4A3BP | Q9Y5P4 | -0.29 | 5.08E-03 | 2.80E-02 |
| MEMO1 | Q9Y316 | 0.21 | 5.19E-03 | 2.85E-02 |
| EBI3 | Q14213 | -0.59 | 5.23E-03 | 2.86E-02 |
| LRRC59 | Q96AG4 | 0.20 | 5.25E-03 | 2.86E-02 |
| NACA | Q13765 | 0.17 | 5.23E-03 | 2.86E-02 |
| PLEK | P08567 | -0.28 | 5.25E-03 | 2.86E-02 |
| RPS12 | P25398 | 0.16 | 5.31E-03 | 2.88E-02 |
| ABCF2 | Q9UG63 | 0.29 | 5.34E-03 | 2.89E-02 |
| RPS19 | P39019 | 0.16 | 5.38E-03 | 2.91E-02 |
| TMED10 | P49755 | 0.24 | 5.40E-03 | 2.91E-02 |
| CSTF3 | Q12996 | 0.17 | 5.49E-03 | 2.95E-02 |
| IGHG1 | P01857 | -1.36 | 5.65E-03 | 3.03E-02 |
| DEK | P35659 | -0.21 | 5.68E-03 | 3.04E-02 |
| FERMT3 | Q86UX7 | -0.10 | 5.72E-03 | 3.04E-02 |
| NAA15 | Q9BXJ9 | 0.13 | 5.71E-03 | 3.04E-02 |
| UQCRC2 | P22695 | 0.11 | 5.68E-03 | 3.04E-02 |
| FDXR | P22570 | -0.27 | 5.76E-03 | 3.05E-02 |
| IRF8 | Q02556 | -0.55 | 5.81E-03 | 3.08E-02 |
| DLST | P36957 | -0.14 | 5.94E-03 | 3.11E-02 |
| SPCS3 | P61009 | 0.28 | 5.91E-03 | 3.11E-02 |
| SURF4 | O15260 | 0.23 | 5.95E-03 | 3.11E-02 |
| TMEM165 | Q9HC07 | 0.21 | 5.89E-03 | 3.11E-02 |
| YARS | P54577 | -0.09 | 5.91E-03 | 3.11E-02 |
| VRK1 | Q99986 | 0.37 | 6.10E-03 | 3.19E-02 |
| BCAT1 | P54687 | -0.70 | 6.20E-03 | 3.23E-02 |
| MARCKS | P29966 | -0.19 | 6.22E-03 | 3.23E-02 |
| TYMP | P19971 | 0.48 | 6.26E-03 | 3.25E-02 |
| LAT2 | Q9GZY6 | 0.44 | 6.29E-03 | 3.26E-02 |
| FEN1 | P39748 | 0.20 | 6.35E-03 | 3.28E-02 |
| NUCKS1 | Q9H1E3 | 0.70 | 6.36E-03 | 3.28E-02 |
| PNPO | Q9NVS9 | 0.37 | 6.37E-03 | 3.28E-02 |
| DDX50 | Q9BQ39 | -0.14 | 6.47E-03 | 3.31E-02 |
| GMPPA | Q96IJ6 | 0.35 | 6.48E-03 | 3.31E-02 |
| POU2F1 | P14859 | -0.29 | 6.47E-03 | 3.31E-02 |
| RAB8B | Q92930 | -0.25 | 6.57E-03 | 3.34E-02 |
| SEC23IP | Q9Y6Y8 | 0.22 | 6.54E-03 | 3.34E-02 |
| ARAP1 | Q96P48 | -0.60 | 6.62E-03 | 3.36E-02 |
| RPS10 | P46783 | 0.23 | 6.70E-03 | 3.39E-02 |
| MESD | Q14696 | 0.27 | 6.73E-03 | 3.40E-02 |
| PFN1 | P07737 | -0.14 | 6.83E-03 | 3.44E-02 |
| ACIN1 | Q9UKV3 | -0.16 | 6.90E-03 | 3.47E-02 |
| KIF2C | Q99661 | -0.38 | 6.89E-03 | 3.47E-02 |
| USP48 | Q86UV5 | 0.27 | 6.92E-03 | 3.47E-02 |
| NCAPG | Q9BPX3 | 0.17 | 7.08E-03 | 3.54E-02 |
| ARHGAP30 | Q7Z6I6 | 0.35 | 7.11E-03 | 3.55E-02 |
| XPO1 | O14980 | 0.07 | 7.31E-03 | 3.64E-02 |
| PSMA6 | P60900 | 0.11 | 7.38E-03 | 3.67E-02 |
| SHMT2 | P34897 | -0.13 | 7.43E-03 | 3.68E-02 |
| NOP2 | P46087 | 0.08 | 7.54E-03 | 3.73E-02 |
| AASDHPPT | Q9NRN7 | 0.28 | 7.62E-03 | 3.76E-02 |
| DHX30 | Q7L2E3 | 0.34 | 7.82E-03 | 3.85E-02 |
| XPO5 | Q9HAV4 | 0.16 | 7.83E-03 | 3.85E-02 |
| AP1B1 | Q10567 | 0.07 | 7.87E-03 | 3.86E-02 |
| PARP1 | P09874 | -0.19 | 7.90E-03 | 3.87E-02 |
| GSS | P48637 | 0.41 | 8.02E-03 | 3.90E-02 |
| HSPB1 | P04792 | -0.44 | 8.04E-03 | 3.90E-02 |
| KARS | Q15046 | 0.07 | 8.00E-03 | 3.90E-02 |
| RALB | P11234 | -0.30 | 8.02E-03 | 3.90E-02 |
| RUFY1 | Q96T51 | -0.38 | 8.02E-03 | 3.90E-02 |
| CCT8 | P50990 | 0.06 | 8.08E-03 | 3.91E-02 |
| NCAPH | Q15003 | 0.15 | 8.09E-03 | 3.91E-02 |
| TRAFD1 | O14545 | -0.54 | 8.14E-03 | 3.92E-02 |
| EIF3C | Q99613 | 0.13 | 8.19E-03 | 3.94E-02 |
| PRKCD | Q05655 | -0.21 | 8.24E-03 | 3.95E-02 |
| NASP | P49321 | -0.23 | 8.42E-03 | 4.03E-02 |
| DNAJB11 | Q9UBS4 | 0.23 | 8.74E-03 | 4.17E-02 |
| KIF11 | P52732 | 0.31 | 8.73E-03 | 4.17E-02 |
| SPTLC1 | O15269 | 0.27 | 8.96E-03 | 4.26E-02 |
| CSTB | P04080 | -0.41 | 8.99E-03 | 4.27E-02 |
| EIF3G | O75821 | 0.11 | 9.06E-03 | 4.29E-02 |
| FLNB | O75369 | -0.32 | 9.14E-03 | 4.31E-02 |
| GSPT1 | P15170 | 0.15 | 9.13E-03 | 4.31E-02 |
| ARF4 | P18085 | 0.14 | 9.39E-03 | 4.41E-02 |
| SF3B1 | O75533 | 0.08 | 9.38E-03 | 4.41E-02 |
| LIG1 | P18858 | 0.45 | 9.42E-03 | 4.42E-02 |
| ARPC2 | O15144 | 0.20 | 9.48E-03 | 4.43E-02 |
| RPAP3 | Q9H6T3 | 0.35 | 9.46E-03 | 4.43E-02 |
| MAT2A | P31153 | 0.19 | 9.53E-03 | 4.45E-02 |
| MGLL | Q99685 | -0.68 | 9.66E-03 | 4.47E-02 |
| RPLP0 | P05388 | 0.24 | 9.65E-03 | 4.47E-02 |
| TRIM22 | Q8IYM9 | 0.20 | 9.60E-03 | 4.47E-02 |
| WDR1 | O75083 | 0.13 | 9.65E-03 | 4.47E-02 |
| PITHD1 | Q9GZP4 | 0.34 | 9.69E-03 | 4.48E-02 |
| BLMH | Q13867 | 0.12 | 9.90E-03 | 4.53E-02 |
| GYS1 | P13807 | -0.42 | 9.83E-03 | 4.53E-02 |
| HNRNPUL1 | Q9BUJ2 | -0.14 | 9.86E-03 | 4.53E-02 |
| RUVBL1 | Q9Y265 | 0.13 | 9.89E-03 | 4.53E-02 |
| SRP19 | P09132 | 0.18 | 9.86E-03 | 4.53E-02 |
| TUBAL3 | A6NHL2 | -0.27 | 1.00E-02 | 4.57E-02 |
| LYAR | Q9NX58 | -0.18 | 1.01E-02 | 4.61E-02 |
| ATXN10 | Q9UBB4 | -0.11 | 1.02E-02 | 4.65E-02 |
| ARHGDIB | P52566 | -0.26 | 1.03E-02 | 4.69E-02 |
| HNRNPD | Q14103 | -0.30 | 1.04E-02 | 4.71E-02 |
| NUCB2 | P80303 | 0.60 | 1.04E-02 | 4.71E-02 |
| PKM | P14618 | 0.09 | 1.04E-02 | 4.71E-02 |
| KPNA3 | O00505 | 0.14 | 1.05E-02 | 4.75E-02 |
| IL4I1 | Q96RQ9 | -0.40 | 1.06E-02 | 4.77E-02 |
| ERLIN2 | O94905 | -0.16 | 1.07E-02 | 4.80E-02 |
| MOB3A | Q96BX8 | -0.39 | 1.07E-02 | 4.80E-02 |
| PABPC4 | Q13310 | 0.20 | 1.09E-02 | 4.86E-02 |
| RABEP1 | Q15276 | -0.26 | 1.09E-02 | 4.86E-02 |
| ATG4B | Q9Y4P1 | 0.18 | 1.10E-02 | 4.89E-02 |
| NCBP1 | Q09161 | 0.15 | 1.10E-02 | 4.89E-02 |
| GSR | P00390 | -0.12 | 1.13E-02 | 4.97E-02 |
| HSPH1 | Q92598 | 0.08 | 1.12E-02 | 4.97E-02 |
| KCNAB2 | Q13303 | -0.26 | 1.12E-02 | 4.97E-02 |
| XRCC5 | P13010 | 0.10 | 1.13E-02 | 4.97E-02 |
