# Supplementary material for: Proteomic investigations of adult polyglucosan body disease: insights into the pathobiology of a neurodegenerative disorder
Source: Front Neurol. 2023 Nov 14;14:1261125. doi: 10.3389/fneur.2023.1261125 (PMC10683643; doi:10.3389/fneur.2023.1261125)
Supplement: Supplementary file 2 [file Table_2.DOCX]

Supplementary Table 2.

Variance in peptide expression of the differentially expressed proteins in the subjects and controls.

|  | Cases (n=3) | Controls (n=15) |
| --- | --- | --- |
| Mean CV % (SD) | 18.18 (14.13) | 18.16 (12.51) |
|  |  |  |
| Lower quartile CV % | 8.21 | 10.92 |
| Median CV % | 14.53 | 14.71 |
| Upper quartile CV % | 23.41 | 21.24 |
