# Supplementary material for: Proteomic investigations of adult polyglucosan body disease: insights into the pathobiology of a neurodegenerative disorder
Source: Front Neurol. 2023 Nov 14;14:1261125. doi: 10.3389/fneur.2023.1261125 (PMC10683643; doi:10.3389/fneur.2023.1261125)
Supplement: Supplementary file 3 [file Table_3.DOCX]

Supplementary Table 3. List of upstream regulators that drive differential expression between APBD cases and controls (p < 1x10^-5^). Predicted activation state describes whether upstream regulator was predicted to be activated or inhibited based on the expression profiles of its downstream targets in cases vs controls. P-values represent one-tailed Fisher’s exact test.

| **Gene** | **Molecule Type** | **Predicted**  **Activation State** | **p-value of**  **overlap** |
| --- | --- | --- | --- |
| MYC | transcription regulator | Activated | 3.53E-24 |
| TP53 | transcription regulator | Inhibited | 4.66E-21 |
| IL4 | cytokine |  | 5.59E-19 |
| XBP1 | transcription regulator | Activated | 1.4E-16 |
| IL3 | cytokine |  | 1.04E-15 |
| MYCN | transcription regulator | Activated | 3.46E-15 |
| NFE2L2 | transcription regulator | Activated | 9.94E-15 |
| HNF4A | transcription regulator |  | 1.49E-14 |
| E2F1 | transcription regulator | Activated | 3.62E-14 |
| CD3 | complex |  | 1.38E-13 |
| MTOR | kinase |  | 1.59E-13 |
| RICTOR | other | Inhibited | 6.73E-13 |
| RRP1B | transcription regulator |  | 8.31E-12 |
| IL5 | cytokine |  | 1.66E-11 |
| CST5 | other |  | 5.85E-11 |
| INSR | kinase |  | 6.69E-11 |
| CD38 | enzyme |  | 7.49E-11 |
| TCR | complex | Activated | 2.15E-10 |
| CD40LG | cytokine |  | 2.55E-10 |
| E2F4 | transcription regulator |  | 2.01E-09 |
| MMP12 | peptidase |  | 4.32E-09 |
| PRL | cytokine |  | 4.4E-09 |
| RB1 | transcription regulator | Inhibited | 3.43E-08 |
| SYVN1 | transporter |  | 0.000000042 |
| CD28 | transmembrane receptor |  | 6.47E-08 |
| IL15 | cytokine |  | 9.97E-08 |
| TGFB1 | growth factor |  | 0.000000171 |
| APP | other |  | 0.000000245 |
| EPO | cytokine |  | 0.000000248 |
| ERBB2 | kinase |  | 0.000000276 |
| ATF6 | transcription regulator |  | 0.000000346 |
| ERN1 | kinase | Activated | 0.000000363 |
| CSF2 | cytokine | Activated | 0.00000042 |
| MAPT | other |  | 0.000000441 |
| KRAS | enzyme |  | 0.000000515 |
| TCF3 | transcription regulator |  | 0.00000082 |
| HTT | transcription regulator |  | 0.00000095 |
| IFNG | cytokine |  | 0.00000101 |
| POLG | enzyme |  | 0.0000017 |
| EIF2AK3 | kinase |  | 0.00000204 |
| ESR1 | ligand-dependent  nuclear receptor | Activated | 0.00000234 |
| TBX2 | transcription regulator | Activated | 0.00000241 |
| HSF1 | transcription regulator |  | 0.0000032 |
| FOLR1 | transporter |  | 0.00000329 |
| HIF1A | transcription regulator |  | 0.00000358 |
| LONP1 | peptidase |  | 0.00000454 |
| AKT1 | kinase |  | 0.0000047 |
| PCGEM1 | other |  | 0.00000486 |
| YY1 | transcription regulator |  | 0.00000527 |
| HRAS | enzyme |  | 0.00000585 |
| WT1 | transcription regulator |  | 0.00000657 |
